# Fast algorithms and heuristics for phylogenomics under ILS and hybridization

**DOI:** 10.1186/1471-2105-14-S15-S6

**Published:** 2013-10-15

**Authors:** Yun Yu, Nikola Ristic, Luay Nakhleh

**Affiliations:** 1 Department of Computer Science, Rice University, Houston, TX, USA

## Abstract

**Background:**

Phylogenomic analyses involving whole-genome or multi-locus data often entail dealing with incongruent gene trees. In this paper, we consider two causes of such incongruence, namely, incomplete lineage sorting (ILS) and hybridization, and consider both parsimony and probabilistic criteria for dealing with them.

**Results:**

Under the assumption of ILS, computing the probability of a gene tree given a species tree is a very hard problem. We present a heuristic for speeding up the computation, and demonstrate how it scales up computations to data sizes that are not feasible to analyze using current techniques, while achieving very good accuracy. Further, under the assumption of both ILS and hybridization, computing the probability of a gene tree and parsimoniously reconciling it with a phylogenetic network are both very hard problems. We present two exact algorithms for these two problems that speed up existing techniques significantly and enable analyses of much larger data sets than is currently feasible.

**Conclusion:**

Our heuristics and algorithms enable phylogenomic analyses of larger (in terms of numbers of taxa) data sets than is currently feasible. Further, our methods account for ILS and hybridization, thus allowing analyses of reticulate evolutionary histories.

## Introduction

The phenomenon of gene tree incongruence arises in phylogenomic studies [1]. This incongruence can be caused by many processes, including incomplete lineage sorting (ILS) and hybridization. Recent studies have shown large extents of gene tree incongruence in various groups of organisms due to ILS [2-9]. Degnan and Salter proposed the first method for computing the probability of a gene tree topology given a species tree using the concept of *coalescent histories *[10]. Later, Wu introduced the concept of *ancestral configurations *(or AC) to speed up the computation for the same task [11], and proposed maximum likelihood method for inference of species tree given a set of gene tree topologies. However, even with the speedup of [11], inference in practice remained limited to data sets with small numbers of taxa, mainly due the very slow computation of gene tree probabilities.

Furthermore, simultaneous patterns of hybridization and incomplete lineage sorting have been reported in several studies [12-14]. Therefore, it is important to enable analyses under both ILS and hybridization. While methods for handling limited cases of reticulation were introduced [15-20], it was not until most recently that parsimony and probabilistic methods for the general cases were introduced [21,22]. However, the methods of [21,22] are computationally intensive and their application is limited to data sets with very few taxa.

The contributions of this paper are threefold. First, we devise a fast heuristic for estimating the probability of a gene tree under ILS. We use the heuristic to infer species trees from multi-locus data, and show that the method achieves significant speedup over the method of [11]. Second, we introduce a novel concept of weighted ancestral configurations on phylogenetic networks and devise a much faster exact algorithm than that of [21] for reconciling a gene tree with a phylogenetic network. Third, using the same concept of weighted ancestral configurations, we devise a much faster exact algorithm than that of [22] for computing the probability of a gene tree under both ILS and hybridization.

We have implemented the methods in the PhyloNet software package [23], which is freely available for download in open source at http://bioinfo.cs.rice.edu/phylonet. The methods will enable much larger phylogenomic analyses than are currently possible using existing methods.

## Methods

Given a directed graph *g*, we denote by *V *(*g*) and *E*(*g*) the node- and edge-sets of *g*. Given a node *v *in a graph *g*, we denote by *d^-^*(*v*), *δ^-^*(*v*), *d*^+^(*v*), *δ*^+^(*v*) the in-degree, in-edges, out-degree, and out-edges, respectively, of node *v*. In this paper, we consider a special type of rooted, directed, acyclic graphs (rDAG) *g *= (*V, E*), where *V *is partitioned into four sets *V_T _*= {*v *∈ *V *: *d^-^*(*v*) = 1 Λ *d*^+^(*v*) ≥ 2}, *V_N _*= {*v *∈ *V *: *d^-^*(*v*) = 2 Λ *d*^+^(*v*) = 1} (reticulation nodes), *V_L _*= *{v *∈ *V *: *d^-^*(*v*) = 1 Λ *d*^+^(*v*) = 0}, and {*r*}, where *r*, the root, is a unique node with *d*^-^(*r*) = 0 and *d*^+^(*r*) ≥ 2. An  X-rDAG is an rDAG whose leaves (the set *V_L_*) are bijectively labeled by set  X. Further, we consider a special type of rooted trees, where a rooted tree is an rDAG with *V_N _*= ∅. An  X-tree is a rooted tree whose leaves are bijectively labeled by set  X.

### Networks and trees

Here, we use the following definition of phylogenetic network topologies [24].

**Definition 1 ***A *phylogenetic  X-network, *or phylogenetic network for short, N is a 3-tuple *(*G, λ, γ*), *where G *= (*V, E*) *is an * X-*rDAG*, λ: *E → *ℝ^+ ^*is the edge lengths mapping, and γ *: *E → *[0, 1] *is the inheritance probability mapping, which satisfies *$\sum_{u\in \delta^{-}\left(v\right)}\gamma \left(u\right)=1$*for every v *∈ *V - *{*r*}.

A *gene tree *on set  Y of alleles (copies of a gene) is a  Y-tree. Since a gene tree is inferred from gene copies that are sampled from a set of species, there is a mapping from the leaves of the gene tree to the leaves of the phylogenetic network.

**Definition 2 ***A species network/gene tree instance is a triplet *(*N, T, µ*), *where N is an * X-*network, T is a * Y-*tree, and *μ:Y→X*is a function that maps the gene tree leaf labels to the phylogenetic network leaf labels*.

Notice that the mapping *µ *is not necessarily injective (as, biologically, not all species have exactly the same number of copies of a given gene) or surjective (since some species may have zero copies of a given gene). In order not to introduce too many symbols, we will refer to the nodes and edges of a phylogenetic network or a gene tree when we actually mean the nodes and edges of the topologies of these structures.

The way in which a gene evolves within the edges of a phylogenetic network can be described by a *coalescent history *[22]. Let *g *be an rDAG or a rooted tree. For a node *u *in *g*, we denote by *g_u _*the set of all nodes that are reachable from node *u*.

**Definition 3 ***Given a species network/gene tree instance *(*N, g, µ*), *a coalescent history is a function h*: *V *(*g*) → *E*(*N *) *that satisfies the following two conditions:*

• *If d*^+^(*v*) = 0 *and µ*(*v*) = *w, then h*(*v*) = (*u, w*) *for the edge *(*u, w*) ∈ *E*(*N *).

• *If v *∈ *g_u _for node u *∈ *V *(*g*), *and h*(*u*) = (*p, q*), *then h*(*v*) = (*x, y*) *where x *∈ *N_q_*.

Given a species network/gene tree instance (*N, g, µ*), we denote by *HN *(*g*) the set of all coalescent histories of *g *given *N*.

### Probability and extra lineages

Two central quantities to compute are *P*(*g|N*), the probability of observing a gene tree topology *g *given the phylogenetic network *N*, and *XL*(*N, g*), the minimum number of extra lineages that arise from the optimal reconciliation of *g *with *N*. We now define these two quantities. The probability of observing gene tree *g *given phylogenetic network *N *is

(1) $$P\left(g|N\right)=\underset{h\in H_{N}\left(g\right)}{\sum }P\left(h|N\right),$$

where

$$P\left(h|N\right)=\frac{w\left(h\right)}{d\left(h\right)}\underset{b\in E\left(N\right)}{\prod }\frac{w_{b}\left(h\right)}{d_{b}\left(h\right)}\gamma {{\left(b\right)}^{ub\left(h\right)}}_{p_{u_{b}(h)vb(h)}}\left(\lambda_{b}\right).$$

In this equation, the entities *u_b_*(*h*) and *v_b_*(*h*) for an edge *b *= (*x, y*) in the phylogenetic network denote the numbers of gene copies that "enter" edge *b *from below (the  Y endpoint) and "exit" edge *b *from above (the *x *endpoint). These two entities define the number of coalescent events that occurred on edge *b*, which equals *r *= *u_b_*(*h*) - *v_b_*(*h*). The probability of *r *coalescent events occurring, reducing *u_b_*(*h*) lineages into *v_b_*(*h*) lineages, on an edge whose length is *λ_b_*, is given by the quantity $p_{u_{b}\left(h\right)v_{b}\left(h\right)}\left(\lambda_{b}\right)$[25]. The quantities *w_b_*(*h*)*/d_b_*(*h*) is the proportion of all *r *coalescent scenarios that are consistent with the gene tree (not every scenario of *r *coalescent events will results in a topology that agrees with *g*) [10]. This quantity without the *b *subscript corresponds to the root of *N *(where there is no explicit edge incoming into it).

Coalescent histories can also be used to compute the minimum number of extra lineages required to reconcile gene tree *g *with *N*. Given a coalescent history *h *: *V *(*g*) → *E*(*N *), the number of extra lineages arising from *h *on an edge *b *= (*w, y*) is *XL*(*b, h*) = max{*vb*(*h*) - 1, 0}. The number of extra lineages arising from *h *on the entire network *N*, denoted by *XL*(*N, h*), is $\sum_{b\in E\left(N\right)}XL\left(b,h\right)$. For a gene tree *g*, the minimum number of extra lineages arising from reconciling it within the edges of phylogenetic network *N*, denoted by *XL*(*N, g*), is

(2) $$XL\left(N,g\right)=\underset{h\in H_{N}\left(g\right)}{\text{min}}XL\left(N,h\right).$$

Notice that for computing extra lineages, the *λ *and *γ *parameters are not used. This is a *maximum parsimony *criterion for explaining the gene tree *g *given the phylogenetic network *N*. Different from Eq. (1), *N *here represents the topology of the network only.

When the phylogenetic network *N *is a tree (that is, *V_N _*= ∅ and *γ*(*b*) = 1, for all *b *∈ *E*(*N *)), algorithms exist for computing *P*(*g|N*) [10,11] and *XL*(*N, g*) [1,26]. More recently, we proposed new methods for computing these two quantities when *N *is a phylogenetic network (*V_N _≠ *∅), based on a technique that converts the network into a multi-labeled tree (MUL-tree) [21,22]. However, these techniques become prohibitive even for data sets with 15 to 20 taxa and even without hybridization being involved [11]. The contribution of this paper, which we present in detail next, is a heuristic that significantly reduces time to approximate *P *(*g|N*), when N is a tree as well as two novel strategies to speed up the algorithms of [21,22] significantly.

### Speeding up probability computation under ILS

From Eq. (1) we can see that the probability of observing gene tree *g *given species tree *N *equals to the sum of all coalescent histories. Number of coalescent histories increases rapidly with the increase of number of taxa, but not all of the histories contribute equally, or even significantly to the sum. We propose the heuristic approach in which the target probability is approximated by summing over a subset of coalescent histories which carry the most weight. In order to do so we first compute limiting coalescent history (*LCH*), which is one distinct, easy to compute, coalescent history and will be used to bound the search space. If *v *is an internal node of species tree *N*, let *b_v _*represent the branch that connects node *v *to its parent with length $\lambda_{b_{v}}$ Let *numL_v _*represent the number of lineages entering branch *b_v _*and *minL_v _*the minimal number of lineages exiting that same branch (if all coalescent events that are permitted by topologies of gene and species tree happen on this branch). Initially all lineages coalesce prior to the root and *numL_v _*for all internal nodes *v *of the species tree is set accordingly. Coalescent history is represented by a vector *C *= (*c*_1_, *c*_2_, ...c*_n-2_*). Each element of *C *corresponds to an internal node (clade) of the gene tree, and the appropriate value represents the node *v *in species tree on whose branch *b_v _*the clade coalesces. *LCH *is just one distinct coalescent history. We define a function called **ComputeLCH **which takes a species tree *N *and gene tree *g*, and returns *LCH *for these two trees.

Algorithm 1: ComputeLCH.

**Input**: Phylogenetic tree *N *with branch lengths, gene tree *g*

**Output**: *LCH(N, g)*

**foreach ***internal node v *∈ *N, with parent w in post-order traversal ***do**

  **if ***minL_v _≠ numL_v _***then**

    Find *x, minL_v _*≤ *x *≤ *numL_v_*, that maximizes $p_{numL_{v},x}\left(\lambda_{v_{b}}\right)$;

    Pick *r *= *numL_v _- x *coalescent events on branch *b_v _*that agree with gene and species tree topologies (ties are broken randomly); Update *LCH *accordingly; *numL_w _← numL_w _- r*;

**return ***LCH*;

Once we have *LCH*, we will approximate the probability of *g *by summing over all coalescent histories that are "under" *LCH*. Coalescent history *H *= (*h*1, *h*2, ...*h_n-2_*) is "under" *LCH *= (*l*_1_, *l*_2_, ..., *l_n-2_*) if for each *i *∈ {1, 2, ..., *n - *2}, *h_i _*is ether equal to *l_i _*or is a descendant of *l_i_*. Similar approach in duplication/loss model was suggested in [27].

We do not address the parsimony approach, since it is trivial when we work with trees. It consists of computing least common ancestor (*LCA*) mapping of all pairs of nodes within a gene tree on a species tree.

### Ancestral configurations on networks

Central to our methods is the concept of weighted *ancestral configuration *(AC, or simply configuration). When its unweighted version was first introduced, it was defined on species trees for computing the probability of gene tree topologies [11]. However, the concept is extended significantly here to apply to networks.

Given a species network *N *with *q *= *|V_N _| *reticulation nodes numbered 1, 2, ..., *q *and a gene tree *g *on set  Y of alleles, an ancestral configuration can be associated with a node *v *of *N*, denoted by *AC_v_*, or an edge *e *of *N*, denoted by *AC_e_*, and is an element of the set $2^{Y}\times {\mathbb{Z}}^{q}\times \mathbb{R}$ where the first element is the set of all subsets of alleles in  Y, the second is the set of all vectors of integers of size *q*, and the third element is the set of real numbers. When the context is clear, we omit the subscript. For an AC (*B, a, w*), the interpretation is as follows:

• *B *⊆ *A*: a set of lineages that exist at the point (node or edge) with which the AC is associated.

• *a*[*i*], for 1 ≤ *i *≤ *q*: an index for the AC split that occurred at reticulation node *i *and gave rise to *B*.

• *w*: a weight of the AC; we discuss below how to set/use this entry.

Given two ACs, *AC*_1 _= (*B*_1_, *a*_1_, *w*_1_) and *AC*_2 _= (*B*_2_, *a*_2_, *w*_2_), we say that *AC*_1 _and *AC*_2 _are *compatible *if for each *i*, 1 ≤ *i *≤ *q*, either *a*_1_[*i*] = *a*_2_[*i*] or *a*_1_[*i*] *· a*_2_[*i*] = 0; otherwise, the two ACs are *incompatible*. Further, if *B*_1 _= *B*_2 _and *a*_1 _= *a*_2_, we say that the two ACs are *identical*.

Ancestral configurations are computed in a bottom-up fashion by algorithms below. Two major operations that occur as the algorithms proceed bottom-up are:

• Splitting an AC whenever a reticulation node is encountered. Let (*B, a, w*) be an AC on the edge incident out of reticulation node *k*. Further, assume that for each reticulation node *i *(1 ≤ *i *≤ *q*), we have a counter *o_i_*, that is initialized to 0 at the start of an algorithm. Splitting (*B, a, w*) at node *k *results in two ACs *AC*_1 _= (*B*_1_, *a*_1_, *w*_1_) and *AC*_2_(*B*_2_, *a_2_, w*_2_), each associated with one of the two reticulation edges, such that *B*_1 _∪ *B*_2 _= *B*, *B*_1 _∩ *B*_2 _= ∅, *a*_1_[*k*] = *a*_2_[*k*] = *o_k _*+ 1, and *o_k _*is incremented by 1. For the weights, *w*_1 _= *w *and *w*_2 _= 0 if the algorithm used is **CountXL **below, and *w*_1 _= *w *and *w*_2 _= 1 if the algorithm used is **CalProb **below.

• Merging two ACs whenever an internal tree node is encountered. Let (*B*_1_, *a*_1_, *w*_1_) and (*B*_2_, *a*_2_, *w*_2_) be two compatible ACs associated with the edges incident from a tree node *u*. Then, these two ACs are merged into one AC (*B, a, w*) at node *u *where *B *= *B*_1 _∪ *B*_2 _and *a*[*i*] = max{*a*_1_[*i*], *a*_2_[*i*]} for all 1 ≤ *i *≤ *q*. For the weights, *w *= *w*_1 _+ *w*_2 _if the algorithm used is **CountXL **below, and *w *= *w*_1 _*· w*_2 _if the algorithm used is **CalProb **below.

For *AC *= (*B, a, w*) we denote by *n*(*AC*) the quantity *|B|*. We denote by AC the set of ACs associated with a node or edge. When AC is associated with an edge, it denotes the set of ACs that result after all coalescence events took place on the edge.

Assume *m *and *n *are two gene lineages that meet at some node in a gene tree *g*. When reconciling *g *within the edges of a species network *N *, after the two entered the same edge of *N *, they might or might not have coalesced before leaving that edge, the probability of which depends on the length (in terms of time) and width (in terms of population size) of that edge. Therefore, one configuration entering a edge of *N *might give rise to several different configurations leaving that edge with different probabilities. We denote by *Coal*(*B, g*), for a set *B *of lineages and gene tree *g*, the set of all sets of lineages that *B *could coalesce into with respect to the topology of *g*. Ancestral configurations provide a compact representation of coalescent histories, thus allowing for efficient computing: while redundant parts that appear in different coalescent histories must be computed explicitly every time they are encountered, particularly over the different allele mappings employed in the approaches of [21,22], using ancestral configurations ameliorates this by computing the values only once for each ancestral configuration. Further, when these computations are coupled with network space search, local perturbations to candidate networks necessitate new computations to only a small number of ancestral configurations. We now show how to use configurations to compute *P *(*g|N*) and *XL*(*N, g*) efficiently.

### Counting the number of extra lineages under ILS and hybridization

For a configuration *AC*, we denote by *xl*(*AC*) the minimum number of extra lineages arising from coalescing the extant gene lineages in *AC *to the present gene lineages in *AC*. In this method, weight *w *in (*B, a, w*) ∈ AC corresponds to *xl*(*AC*), where AC is either ${AC}_{v}$ where *v *is a node or ${AC}_{b}$ where *b *is a edge.

**Observation 1 ***Let AC *= (*B, a, w*) *be a configuration entering a edge b and AC*^+ ^= (*B*^+^, *a*^+^, *w*^+^) *be a configuration that AC coalesced into before leaving b. Then w*^+ ^= *w*^+ ^max{*n*(*AC*^+^) - 1, 0}, *where n*(*AC*^+^) *is the number of lineages on edge b*.

We define a function called **CreateCACsForXL **which takes a gene tree *g*, an edge *b *= (*u, v*) of the network *N *and a set of ACs ${AC}_{v}$ that enter edge *b*, and returns a set of ACs ${AC}_{\left(u,v\right)}$ that exit edge *b*.

Note that although one configuration can coalesce into several different configurations along an edge, under parsimony we only need to keep the one that has the minimum total number of extra lineages. Therefore

Algorithm 2: CreateCACsForXL.

**Input**: Gene tree *g*, edge *b *= (*u, v*), set of ACs ${AC}_{v}$

**Output**: A set of ACs ${AC}_{\left(u,v\right)}$

**foreach **(*B, a, w*) ∈ ${AC}_{v}$**do**

  $B^{+}\leftarrow \text{argmi}{\text{n}}_{B^{\prime }\in Coal\left(B,g\right)}|B^{\prime }|;$

  Compute *w*^+ ^using Rule 1;

  ${AC}_{\left(u,v\right)}\leftarrow {AC}_{\left(u,v\right)}\cup \left(B^{+},\;a\;w^{+}\right)$;

**return **${AC}_{\left(u,v\right)}$

$\left|{AC}_{u}|=|{AC}_{\left(u,v\right)}\right|$ and there is 1-1 correspondence between configurations in $\left|{AC}_{u}\right|$ and configurations in $\left|{AC}_{\left(u,v\right)}\right|$.

For a phylogenetic network *N *and a gene tree *g*, the algorithm for computing the minimum number of extra lineages required to reconcile *g *within *N *is shown in Alg. 3. Basically, we traverse the nodes of the network in post-order. For every node *v *we visit, we construct the set of ACs ${AC}_{v}$ for node *v *based on its type. Recall that there are four types of nodes in a phylogenetic network, which are leaves, reticulation nodes, internal tree nodes, and the root. Finally when we arrive at the root of *N*, we are able to obtain *XL*(*N, g*).

Algorithm 3: CountXL.

**Input**: Phylogenetic network *N *with *q *reticulation nodes, gene tree *g*

**Output**: *XL*(*N, g*)

**while ***traversing the nodes of N in post-order ***do**

**if ***node v is a leaf, who has parent u ***then**

  ${AC}_{v}\leftarrow \left\{\left(B,\;a\;0\right)\right\}$ where *B *is the set of leaves in *g *that are sampled from the species associated with *v *and *a *is a

  vector of *q *0's;

  ${AC}_{\left(u,v\right)}$ ← CreateCACsForXL(*g*, (*u, v*), ${AC}_{v}$);

**else if ***node v is a reticulation node, who has child w, and two parents u*_1 _*and u*_2 _**then**

  ${AC}_{v}\leftarrow {AC}_{\left(v,w\right)}$;

  **foreach ***AC *∈ ${AC}_{v}$**do**

    Split *AC *in every possible way into pairs of ACs, and for each pair, add one AC to ${AC}_{\left(u_{1},v\right)}$ and the other AC to ${AC}_{\left(u_{2},v\right)}$;

**else if ***node v is an internal tree node, who has two children w*_1 _*and w*_2 _**then**

  **foreach ***pair *(*AC*_1_, *AC*_2 _) *of compatible ACs in *${AC}_{\left(v,w_{1}\right)}\times {AC}_{\left(v,w_{2}\right)}$**do**

    Merge *AC*_1 _and *AC*_2 _and add the resulting AC to ${AC}_{v}$;

  **if ***node v is an internal tree node, who has a parent u ***then**

    ${AC}_{\left(u,v\right)}$ ← CreateCACsForXL(*g*, (*u, v*), ${AC}_{v}$);

  **else**

    **return **${\text{min}}_{AC\in {AC}_{v}}\;xl\left(AC\right)$;

### Calculating gene tree probability under ILS and hybridization

For a configuration *AC*, we denote by *p*(*AC*) the cumulative probability of the extant gene lineages in *AC *coalescing into the present gene lineages in *AC *from time 0. In this method, weight *w *in *AC *= (*B, a, w*) ∈ AC corresponds to *p*(*AC*), where AC is either ${AC}_{v}$ where *v *is a node or ${AC}_{b}$ where *b *is an edge.

**Lemma 1 ***Let B be a set of gene lineages entering branch b of network N with branch length λ_b_. Then the probability of observing a set of gene lineages B*^+ ^*leaving branch b is*

(3) $$p_{t}\left(B,\;B^{+},\;b\right)=p_{|B|,|B+}|\left(\lambda_{b}\right)\frac{w_{b}\left(B,B^{+}\right)}{d_{b}\left(B,B^{+}\right)}$$

*where *$p_{|B|,|B+}|\left(\lambda_{b}\right)$*is the probability that |B| gene lineages coalesce into |B*^+^*| gene lineages within time λ_b_, wb*(*B, B*^+^) *is the number of ways that coalescent events can occur along edge b to coalesce B into B*^+ ^*with respect to the gene tree topology, and db*(*B, B*^+^) *is the number of all possible orderings of |B|-|B*^+^*| coalescent events*.

**Observation 2 ***Let AC *= (*B, a, w*) *be a configuration entering an edge b and AC*^+ ^= (*B*^+^, *a*^+^, *w*^+^) *be a configuration that AC coalesced into when leaving b. Then w*^+ ^= *w · p_t_*(*B, B*^+^, *b*).

We define a function called **CreateCACsForProb **which takes a gene tree *g*, an edge *b *= (*u, v*) of the network *N *and a set of ACs ${AC}_{v}$ that enter edge *b*, and returns a set of all possible ACs ${AC}_{\left(u,v\right)}$ that exit edge *b*. The algorithm for calculating the probability of observing a gene tree *g *given a species network *N*

Algorithm 4: CreateCACsForProb.

**Input**: Gene tree *g*, an edge *b *= (*u, v*), a set of ACs ${AC}_{v}$

**Output**: A set of ACs ${AC}_{\left(u,v\right)}$

**foreach **(*B, a, w*) ∈ ${AC}_{v}$**do**

  **foreach ***B*^+ ^∈ *Coal*(*B, g*) **do**

    Compute *w*^+ ^using Rule 2;

    **if **∃(*B*', *a*', *w*') ∈ ${AC}_{\left(u,v\right)}$*where B*' = *B*^+ ^*and a*' = *a ***then**

      *w*' ← *w*' + *w*^+^;

    **else**

      ${AC}_{\left(u,v\right)}\leftarrow {AC}_{\left(u,v\right)}\cup \left(B^{+},\;a\;w^{+}\right)$;

**return **${AC}_{\left(u,v\right)}$;

is shown in Alg. 5. The basic idea is similar to the parsimony method we described in the previous section. It is important to note that the running time of the algorithms can be exponential for some data sets, as the complexity of both problems is open and conjectured to be NP-hard.

### Reducing the number of configurations

At every reticulation node *v *in the species network, every configuration *AC *in ${AC}_{v}$ is split in all 2^*n*(*AC*) ^possible ways. This may result in multiple configurations which contain the same set of gene lineages but are all distinct because of different vector values in some AC. It is clear that the running time (and memory usage) of both these two algorithms depends on the number of configurations. Therefore, in order to reduce the number of configurations so as to speed up the computation, we make use of *articulation*

Algorithm 5: CalProb.

**Input**: Phylogenetic network *N *including topology, edge lengths and inheritance probabilities, gene tree *g*

**Output**: *P *(*g|N*)

**while ***traversing the nodes of N in post-order ***do**

  **if ***node v is a leaf, whose parent is u ***then**

    ${AC}_{v}\leftarrow \left\{\left(B,\;a\;1\right)\right\}$ where *B *is the set of leaves in *g *sampled from the species associated with *v *and *a *is a vector of *q *0's;

    ${AC}_{\left(u,v\right)}$ ← CreateCACsForProb(*g*, (*u, v*), ${AC}_{v}$);

  **else if ***node v is a reticulation node, who has child w, and two parents u*_1 _*and u*_2 _**then**

    ${AC}_{v}\leftarrow {AC}_{\left(v,w\right)}$;

    *S*_1 _← ∅;

    *S*_2 _← ∅;

    **foreach ***AC *∈ ${AC}_{v}$**do**

      Split *AC *in every possible way into pairs of ACs, and for each pair, add one AC to *S*_1 _and the other AC to *S*_2_;

    **foreach **(*B, a, w*) ∈ *S*_1 _**do**

      $w\leftarrow w\cdot \gamma_{\left(u_{1},v\right)}^{\left|B\right|};$

    ${AC}_{\left(u_{1},v\right)}$ ← CreateCACsForProb(*g*, (*u*_1 _, *v*), *S*_1_);

    **foreach **(*B, a, w*) ∈ *S*_2 _**do**

      $w\leftarrow w\cdot \gamma_{\left(u_{2},v\right)}^{\left|B\right|};$

    ${AC}_{\left(u_{2},v\right)}$ ← CreateCACsForProb(*g*, (*u*_2 _, *v*), *S*_2 _);

  **else if ***node v is an internal tree node, who has two children w*_1 _*and w*_2 _**then**

    **foreach ***pair *(*AC*_1_, *AC*_2_) *of compatible ACs in *${AC}_{\left(v,w_{1}\right)}\times {AC}_{\left(v,w_{2}\right)}$**do**

      Merge *AC*_1 _and *AC*_2 _and add the resulting AC to ${AC}_{v}$;

    **if ***node v is an internal tree node, who has a parent u ***then**

      ${AC}_{\left(u,v\right)}$ ← CreateCACsForProb(*g*, (*u, v*), ${AC}_{v}$);

    **else**

      Let *B_R _*be the root lineage of the gene tree *g*;

      **return **$\sum_{\left(B,a,w\right)\in {AC}_{v}}w\cdot p_{t}\left(B,\;B_{R},+\infty \right)$;

nodes in the graph (an articulation node is a node whose removal disconnects the phylogenetic network). Obviously, the reticulation nodes inside the sub-network rooted at an articulation node are independent of the reticulation nodes outside the sub-network. So at articulation node *v *we can reset the vectors in all ACs in ${AC}_{v}$ to 0's so that all configurations at *v *containing the same set of gene lineages become identical. More precisely, when traversing the species network, after constructing ${AC}_{v}$ for some internal tree node *v *as we have described in Alg. 3 and Alg. 5, if *v *is an articulation node, we reset the vector to 0's in every AC in ${AC}_{v}$. Then for counting the minimum number of extra lineages, we update ${AC}_{v}$ to be ${AC}_{v}^{\prime }$ such that only the configuration containing the minimum weight is left, using the statement: ${AC}_{v}^{\prime }=\left\{{\text{argmin}}_{\left(B,a,w\right)\in {AC}_{v}}w\right\}$. And for computing the probability of the topology of a gene tree, we keep only one copy of every distinct configuration. More precisely, we update ${AC}_{v}$ to be ${AC}_{v}^{\prime }$ using ${AC}_{v}^{\prime }=\left\{\left(B,a,w^{\prime }\right):w^{\prime }=\sum_{\left(B,a,w\right)\in {AC}_{v}}w\right\}$. Note that *a *is a zero vector.

## Results and discussion

### Species tree inference under ILS

It is important to note that although we used coalescent histories to describe the heuristic, in practice we estimate the probability of the gene tree given a species tree using the concept of ancestral configurations.

To test the speed of proposed heuristic we generated 4 datasets, each consisting of 50 random species trees with same number of taxa and same height using PhyloGen [28]. We used trees with 10, 20, 30 and 300 taxa of heights 5, 10, 15 and 20 respectively. From each species tree we simulated 25 gene trees, and estimated the probability of the gene trees given that species tree. On average, to compute (or estimate) the probability of a single gene tree, for the exact method it took 0.0019, 0.297 and 10.05 and for heuristic 0.0019, 0.0068 and 0.01 seconds for the trees with 10, 20 and 30 taxa, respectively. For the exact method we see that as we increase the number of taxa the time starts increasing dramatically. For the dataset consisting of trees with 300 taxa using heuristic took on average 0.2594 seconds to compute the same probability.

**Figure 1:**
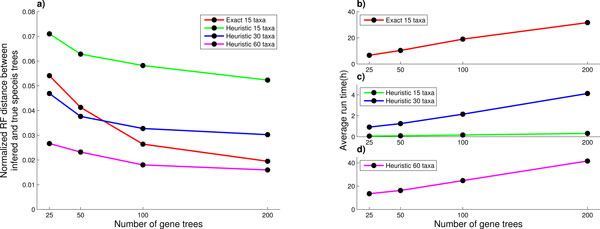
**Normalized RF distance between inferred and true species trees (a), and average running times (b), (c) and (d) for heuristic with 15, 30 and 60 taxa and exact method for 15 taxa**.

To further test the proposed heuristic we modified and ran the maximum likelihood species tree inference program STELLS [11] on synthetic data generated by PhyloGen. Modification of STELLS consisted of changing the function that computes the probability of the gene tree given a species tree to use the proposed heuristic. Tests were performed on 3 different datasets each consisting of 10 randomly generated species trees. For the first dataset all trees contained 15 taxa and were of height 7 coalescent units. On this data set we ran both unmodified STELLS (which uses all coalescent histories to compute the probability of the gene tree given a species tree) and modified STELLS which employs the proposed heuristic. For the second dataset all trees contained 30 taxa and were of height 15 coalescent units. For the third dataset all trees contained 60 taxa and were of height 30 coalescent units. From each of the species trees we have generated 120 sets consisting of 30 sets with 25 gene trees, 30 sets with 50 gene trees, 30 sets with 100 gene trees and 30 sets with 200 gene trees. We have imposed the 72 hour time constrain on execution time, that is, killed the jobs that did not complete in 72 hours. We compared the inferred species tree with the true species tree (the tree from which we simulated the gene trees) in terms of averaged normalized Robinson-Foulds (RF) distance [29], Figure 1(a), as well as the average inference runtime, Figure 1 (b-d).

For the inference using heuristic on datasets with 15 and 30 taxa all of the runs finished within the given time frame of 72 hours. On the dataset with 60 taxa 5 out of 300 runs with 25 gene trees, 6 out of 300 runs with 100 gene trees and 31 out of 300 runs with 200 gene trees did not finish in the given time frame. For the exact method on 15 taxa data set, 1 out of 300 runs with 100 gene trees and 16 out of 300 runs with 200 gene trees did not finish in the given time frame.

From Figure 1(a) we can see that if we have more gene trees the inferred species tree will be more accurate, which is a good property. When the heuristic is compared with exact method on 15 taxa dataset, the accuracy of the heuristic decreases slightly, but the running time improves significantly. Average speedup of the heuristic over the exact method on this dataset was 112.

One important thing to keep in mind while looking at the runtime results is that we are working with ML framework. In order to infer species tree we need to examine large number of potential species trees, for each of them we need to estimate branch lengths and finally compute the likelihood function. This means that for any potential species tree we will have to recompute the probabilities of all given gene trees multiple times. So when we increase the number of taxa, we increase the number of potential species tree we have to evaluate, and we increase the number of branches within the tree. This is the reason why the computation on datasets with large number of taxa will still take long time, but not due to the slow computation of probability.

### Gene tree parsimonious reconciliation and probability under ILS and hybridization

To study the performance of the two methods compared to the MUL-tree based methods, we ran all four on synthetic data generated as follows. We first generated 100 random 24-taxon species trees using PhyloGen [28], and from these we generated random species networks with 1, 2, 4, 6 and 8 reticulation nodes. When expanding a species network with *n *reticulation nodes to a species network with *n *+ 1 reticulation nodes, we randomly selected two existing edges in the species network and connected their midpoints from the higher one to the lower one and then the lower one becomes a new reticulation node. Then, we simulated 10, 20, 50, 100, 200, 500 and 1000 gene trees respectively within the edges of each species network using the ms program [30]. Since the MUL-tree methods are computationally very intensive, we employed the following strategy: for the parsimony methods, we bounded the time at 24 hours (that is, killed jobs that did not complete within 24 hours). For the probabilistic methods, we bounded the time at 8 hours. The reason for this choice is that we found that in most cases if the probabilistic method did not finish within 8 hours, then it often did not finish within 24 hours (which is *not *the case of the parsimony methods). Therefore, to save running time that would be "wasted" without adding to the results, we decided on the 8-hour bound for the probabilistic methods.

**Figure 2:**
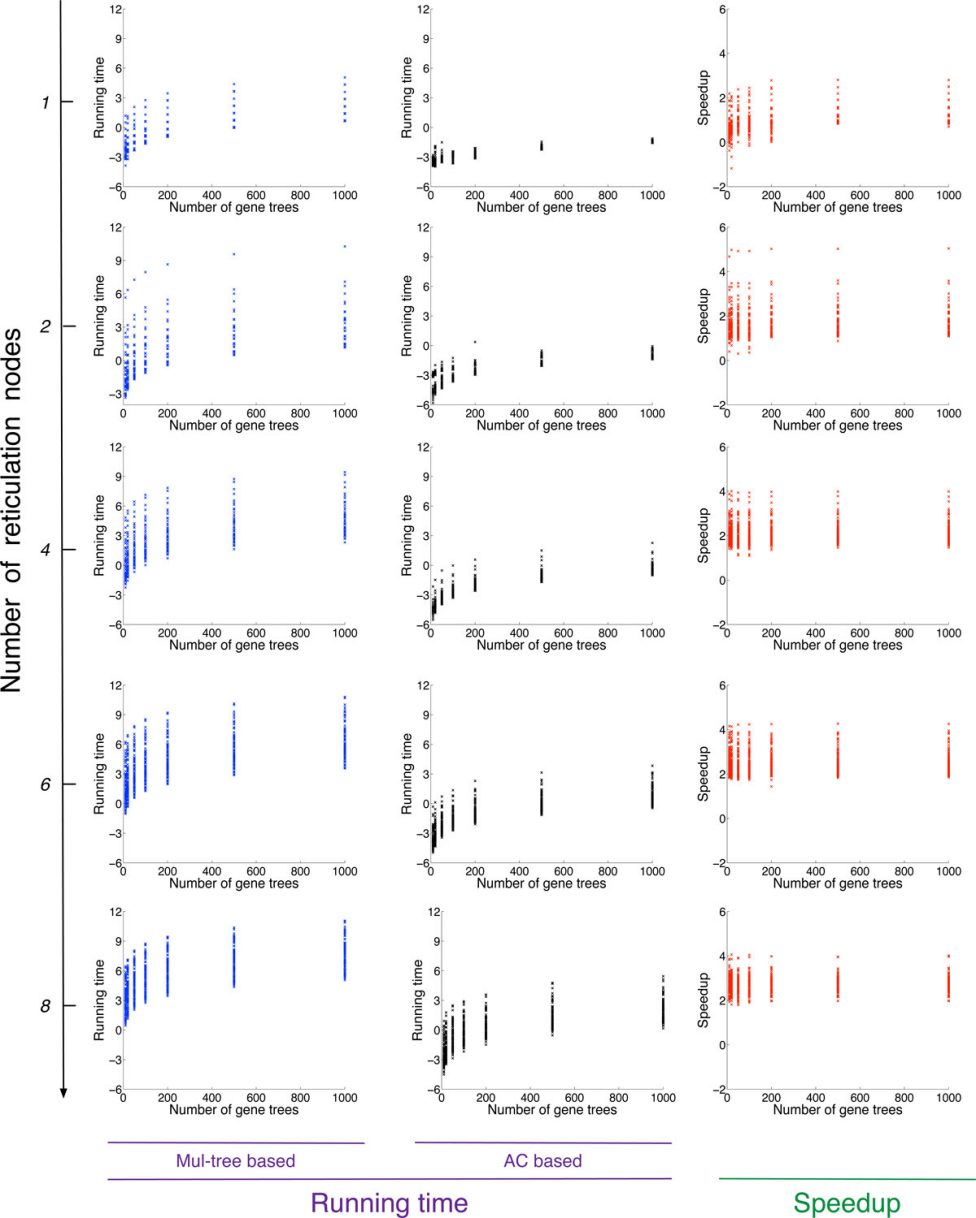
**The running times (ln of number seconds) of the MUL-tree based (*t*(*MUL*)), and AC-based (*t*(*AC*)) methods for computing parsimonious reconciliations, as well as the speedup *log*_10_(*t*(*MUL*)*/t*(*AC*))**.

For computing the minimum number of extra lineages, the results of the running time of both methods are shown in Figure 2. Overall, both methods spent more time on data sets where the species networks contain more reticulation nodes. It is not surprising given the fact that adding more reticulation nodes increases the complexity of the networks in general. We can see that the speedup of the AC-based method over the MUL-tree based method also increased when the number of reticulation nodes in the species networks increased. In the best cases, the method achieves an improvement of about 5 orders of magnitude. In this figure, we only plot the results of the computations that could finish in 24 hours across all different number of loci sampled. In fact, the AC based method finished every computation in less than 3 minutes, even for the largest data set which contained species networks with 8 reticulations and 1000 gene trees. For the MUL-tree based method, out of 100 repetitions the numbers of repetitions that were able to finish in 24 hours across all different loci are 100, 100, 99, 96 and 88 for data sets containing species networks with 1, 2, 4, 6 and 8 reticulation nodes.

**Figure 3:**
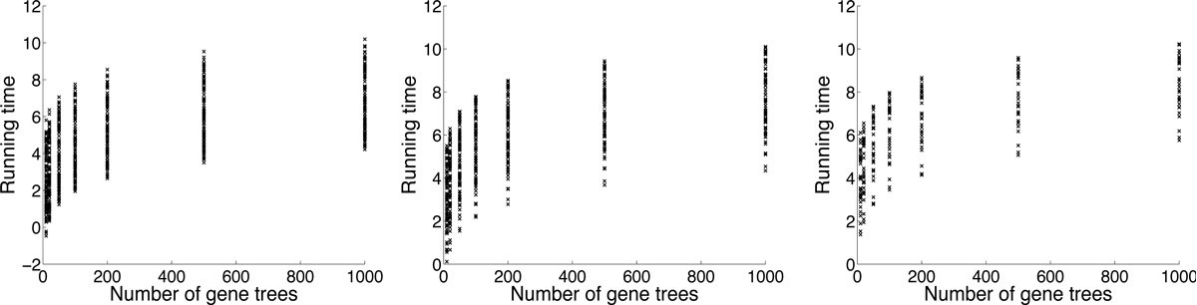
**The running time (ln of number of seconds) of the AC-based algorithm for computing the probability of gene tree topologies given a species network**. The columns from left to right correspond to data sets containing species networks with 1, 4 and 8 reticulation nodes, respectively.

For computing the probability of the gene tree topologies given a species network, we were not able to run the MUL-tree based method because we found it could not finish the computation in 24 hours even for the smallest data set (one gene tree and a species network with one reticulation node). In contrast, the AC-based method only needed 0.4 seconds on the same data set which implies a speedup of at least 5 orders of magnitude. Part of the results of the AC based algorithm are shown in Figure 3.

For both parsimony and probabilistic methods, it is not surprising to see that for a fixed number of taxa the running time increases significantly when the number of reticulation nodes in the species networks increased. However, even for the same number of reticulation nodes, we can see that the running time still differs significantly from case to case. We find that for the data sets of the same size (e.g., number of taxa and reticulation nodes) there are several factors that can affect the number of configurations generated during the computation which directly dominates the running time of the algorithm. More specifically, the running time of the algorithms increases when there are more leaves under reticulation nodes and when the reticulation nodes are more dependent on each other. With respect to the topology of the gene tree and the species network, the more coalescent events that are allowed under reticulation nodes the faster the parsimony method is, and the opposite for the probabilistic method. For most cases, the AC-based methods are significantly much faster than the MUL-tree based ones. For parsimony, the gain in terms of efficiency comes from avoiding allele mappings that are guaranteed to result in suboptimal reconciliations or correspond to configurations being removed at articulation nodes. For probabilistic reconciliation, the gain comes from two factors: (1) avoiding redundant computations by reducing the number of configurations at articulation nodes which could not be avoided in MUL-tree based method, and (2) using ACs to compute the probability instead of enumerating the coalescent histories.

## Competing interests

The authors declare that they have no competing interests.

## Declarations

Publication of this article was funded in part by NSF grants DBI-1062463 and CCF-1302179, grant R01LM009494 from the National Library of Medicine, an Alfred P. Sloan Research Fellowship, and a Guggenheim Fellowship to L.N. This work was also supported in part by the Data Analysis and Visualization Cyberinfrastructure funded by NSF under grant OCI-0959097. The contents are solely the responsibility of the authors and do not necessarily represent the official views of the NSF, National Library of Medicine, the National Institutes of Health, the Alfred P. Sloan Foundation, or the John Simon Guggenheim Memorial Foundation.

This article has been published as part of *BMC Bioinformatics *Volume 14 Supplement 15, 2013: Proceedings from the Eleventh Annual Research in Computational Molecular Biology (RECOMB) Satellite Workshop on Comparative Genomics. The full contents of the supplement are available online at http://www.biomedcentral.com/bmcbioinformatics/supplements/14/S15.
